# The Alcoholization of Wines

**Published:** 1887-03

**Authors:** 


THE ALCOHOLIZATION OF WINES.—In the season of the Academy of Medicine, of France, held November 30, 1886, the Academy discussed at length the question of the reinforcing of wines with alcohol, and, after a long controversy, arrived at the following conclusions:

1. The addition of pure alcohol to wines in quantities not exceeding two degrees may be allowed; but amount greater than this cannot be tolerated.

2. Addition of alcohol is dangerous, not only because of the quantity and quality of poor alcohol used, but because it destroys confidence and perpetuates fraud.

3. Alcohols commonly called good increase considerably the injurious effects of brandy and liqueurs; alcohol added to these liquids should be absolutely pure.

4. The Academy called the attention of the authorities to the necessity of reducing the number of smaller warehouses for keeping and altering wines, and of putting in active operation laws against drunkenness.—*Bulletin de l'Académie de Médecine*, No. 48.

